# Through the eyes of nurses: a content analysis of nurses’ experiences in caring for COVID-19 patients

**DOI:** 10.1186/s12912-023-01601-5

**Published:** 2023-11-15

**Authors:** Razieh Beigi Broujeni, Hesamaddin Kamalzadeh, Zakieh Ahmadi, Samireh Abedini

**Affiliations:** 1 Department of Nursing, School of Nursing and Midwifery, Mother and Child Welfare Research Center, Hormozgan University of Medical Science, Bandar Abbas, Iran; 2 Department of Health Information Technology, School of Paramedical Sciences, Hormozgan University of Medical Sciences, Bandar Abbas, Iran; 3 Department of Nursing, School of Nursing and Midwifery, Hormozgan University of Medical Science, Bandar Abbas, Iran; 4 Department of Medical Education, School of Medicine, Hormozgan University of Medical Sciences, Bandar Abbas, Iran

**Keywords:** Nurses, COVID-19, Patients, Qualitative research

## Abstract

**Background:**

In the current global climate, the nursing staff has placed a significant emphasis on developing effective self-protection strategies and implementing measures to prevent the transmission of infectious diseases, with a particular focus on the highly communicable COVID-19 virus. Ensuring the safety and well-being of both healthcare providers and patients has made it imperative to incorporate this aspect into healthcare provision.

**Objective:**

The study aimed to describe the experiences of nurses in providing care for patients infected with COVID-19.

**Methodology:**

This qualitative content analysis study, following COREQ guidelines, involved 18 nurses who were taking care of COVID-19 patients at Shahid Mohamadi Hospital, a general hospital in Bandar Abbas, Hormozgan Province. The hospital is affiliated with Hormozgan University of Medical Sciences and the research was conducted in 2022.The sampling method was purposive, and unstructured interviews were used. Data collection continued until data saturation was achieved. Data analysis was performed continuously and concurrently through the collection of data using conventional content analysis methods.

**Findings:**

The qualitative analysis of the data revealed three main themes centered on challenges related to nurses’ self-care, patient care, and the healthcare delivery system.

**Conclusion:**

Overall, to address the challenges faced by healthcare providers caring for COVID-19 patients, their needs must be prioritized. This includes establishing a prepared healthcare system, implementing strategies to protect their well-being, and learning from their experiences for future disease outbreaks and disasters.

**Supplementary Information:**

The online version contains supplementary material available at 10.1186/s12912-023-01601-5.

## Background

The outbreak of the novel Coronavirus originated in China in late 2019 [1]. Initially, the infection rate was relatively low [2]. However, by January 2020, the disease started spreading to other countries, capturing global attention [3]. On February 11, 2020, the World Health Organization (WHO) officially named the new virus COVID-19 [4]. Simultaneously, the international virus classification committee changed the name from 2019-nCoV to SARS-CoV-2 [5]. Recognizing the escalating infection rate and global spread, the WHO declared COVID-19 a global public health emergency on January 30, 2020 [4]. As of September 24, 2023, there have been a total of 771 million confirmed cases worldwide, with over 6 million reported fatalities [6]. Hence, the disease is regarded as highly contagious and deadly, with considerable health and psychosocial effects on numerous societal groups [7]. Currently, there is no definitive antiviral treatment for the disease, and the focus remains on patient care and minimizing adverse effects such as secondary infections and organ failure [3].

Healthcare providers are among the primary resources of a country, and their health or immunity is of an utmost importance for the consistent care-taking of patients and controlling the pandemics [8]. The highly infectious nature of the COVID-19 virus puts healthcare providers, including nurses, at a heightened risk of contracting the disease [9, 10]. Nurses, being the largest group of healthcare professionals globally, play a critical role in improving the quality of healthcare services [11, 12]. They deliver at least 50% of health services and, in some countries, are responsible for up to 80% of healthcare procedures, underscoring their substantial contribution [13]. As frontline workers, nurses are crucial in addressing the pandemic while also needing to prioritize their well-being. However, it is important to recognize the significant challenges that nurses face in fulfilling their critical responsibilities during the COVID-19 pandemic. In response to the global situation, the nursing staff has prioritized the development of effective self-protection strategies and the implementation of measures to prevent the transmission of highly contagious diseases, specifically the COVID-19 virus [14].

There have been numerous studies investigating the impact of COVID-19 on nurses who are actively involved in caring for patients with the virus [7, 10, 15–21]. These studies provide evidence that nurses face a wide range of challenges that significantly affect their overall well-being. These challenges include the fear and stress associated with the risk of contracting and transmitting the virus, the uncertainty resulting from constantly evolving protocols, and the physical exhaustion caused by heavy workloads and the use of personal protective equipment. It is crucial to note that a lack of proper infection control or sufficient personal protective equipment can jeopardize essential healthcare services in the fight against the virus [22]. Additionally, the increasing number of suspected or confirmed cases, high work pressure, absence of a definitive cure, and perceived lack of support all contribute to mounting mental pressure on healthcare staff [23]. Addressing these challenges is vital as nurses play a critical role as primary caregivers, and any obstacles they encounter can potentially impact the entire patient care system [24].

It is important to consider that in Iran, the majority of hospitals and healthcare centers are under the ownership and administration of the national healthcare system, which is overseen by the Ministry of Health, Treatment & Medical Education. Nurses constitute approximately 80% of the healthcare workforce, with a significant portion employed in public hospitals. Additionally, nurses play a crucial role by performing about 80% of the tasks within hospitals [25, 26]. As of the WHO’s situation report on October 22nd, 2023, Iran has reported a total of 7,619,981 confirmed COVID-19 cases and 146,480 deaths related to the virus [27]. These numbers reflect the severe impact of the COVID-19 pandemic on Iran’s population. In view of the above, it is important to note that there is still a limited number of qualitative studies specifically focused on nurses’ experiences in caring for patients with COVID-19 [19, 25, 26, 28, 29]. Qualitative research on nurses’ experiences during the COVID-19 pandemic is a developing field that is producing a growing body of evidence [30]. Recent qualitative studies have provided valuable insights into the psychological challenges faced by nurses caring for patients infected with COVID-19 in Iran. These studies have shed light on the psychological challenges, including stress and fear of infection [25, 26, 29], as well as the social aspects such as social panic and the need for improved social support [19, 29]. Furthermore, organizational challenges related to improper planning, difficulties with protocols and guidelines, and a lack of personal protective equipment have been identified [26, 29]. Additionally, professional challenges such as inadequate staff training and unreasonable work shifts have been reported by nurses in this context [26, 28, 29]. Taking into account the lack of research in Hormozgan province regarding nurses’ experiences in caring for COVID-19 patients, and recognizing the potential variations in these experiences due to differences in resources and cultural contexts across hospitals, a qualitative content analysis method, commonly employed in nursing science research, was utilized [31]. Hence, the aim of this study was to describe the experiences of nurses in providing care for patients infected with COVID-19.

## Methods

### Study design

This study was conducted as a qualitative investigation, employing the qualitative content analysis approach. This approach, which is commonly utilized in nursing science research [31], is well-suited for extracting meaning and comprehending the lived experiences of study participants [32]. The study data was reported in accordance with the guidelines outlined in the Consolidated Criteria for Reporting Qualitative Research (COREQ) checklist [33].

### Participant selection

Front-line nurses, specifically those directly involved in providing care to COVID-19 patients, were selected using a purposive sampling method. The inclusion criteria for this study were as follows: participants were required to hold a bachelor’s degree (or higher) in nursing, work as front-line nurses, have a minimum of 2 years of direct experience in caring for COVID-19 patients, and express willingness to participate in the study. To recruit eligible participants, a list of nurses who met the inclusion criteria was obtained from the nursing administrative office. Potential participants were provided with information regarding the study’s purpose, ethical considerations (including the right to withdraw at any stage), data confidentiality, and anonymity. Following this, they were invited to participate in face-to-face interviews. The sample size was determined by applying the concept of data saturation, which refers to the stage where no new themes or codes emerge from the data, indicating information redundancy [34]. In this study, data saturation was achieved after conducting 18 interviews.

### Setting

This study was conducted at Shahid Mohammadi Hospital, which is an affiliated teaching hospital for Hormozgan University of Medical Sciences in Bandar Abbas, Iran. The hospital is a multi-specialty facility located in the Hormozgan Province and serves as a referral center. In order to enhance the generalizability of the findings, the participants were chosen from a varied range of age groups, genders, and nursing work experiences. The interviews with participating nurses were conducted during the period of November to December 2022, at a time mutually agreed upon within the hospital setting. There were no other individuals present during the interviews.

### Data collection

Data were collected through unstructured interviews. The face-to-face interviews were organized and conducted with nurses at the end of their work shifts in a private room at the hospital, ensuring privacy and minimizing interruptions. Before the interviews began, the purpose of the study was explained, and written informed consent was obtained from each nurse in accordance with ethical guidelines. The interview process was explained, and participants were asked to provide information about their age, gender, education, and work experience. Open-ended questions were then used to initiate the interviews, which were recorded using an audio electronic device. None of the nurses refused to participate or withdrew from the study. The main questions of the study were: “Could you please share your experience in caring for patients with COVID-19?“ The interview process involved asking more detailed questions based on the participants’ responses, such as “What were the biggest challenges you faced while caring for COVID-19 patients?“, “What precautions did you take to protect yourself and others from COVID-19 while caring for patients?“, and “What types of confusion and uncertainty arose when caring for COVID-19 patients?“. The interviews lasted 60–90 min per person. Following each interview, the content was transcribed and shared with the participants to ensure the accuracy of the recorded information. Data saturation, where no new themes emerged, was achieved after 18 interviews.

### Data analysis

The data collection and analysis were performed simultaneously, with all interviews being recorded in audio format and transcribed promptly for analysis. The content analysis approach developed by Graneheim and Lundman [35] took place in five steps: (1) reading the entire text several times to gain an overall understanding of its content; (2) breaking it up into meaningful units and condensing them; (3) abstracting the condensed meaning units; (4) reviewing the abstracted meaning units in light of the study’s purpose; and (5) comparing and organizing the meaning units into themes and subthemes.

## Results

The study involved interviewing a total of 18 participants, consisting of two males and sixteen females, who had an average of 8.5 years of nursing experience. Among the participants, three had previous managerial experience as head nurses. Table 1 provides an overview of the characteristics of the nurses who were interviewed. On average, the interviews lasted for 75 min. The data analysis process yielded 186 primary codes, which were categorized into three main themes and ten subthemes. These main themes centered on challenges related to nurses’ self-care, patient care, and the healthcare delivery system, as detailed in Table 2. **Nurses’ self-care challenges**.

**Table 1:** Nurses characteristics (N = 18)

Characteristics	N
Gender	
Female Male	16 2
Education	
Bachelor Master	14 4
Age (range 26–46 years)	Average
Female Male	32.7 35
Work experience (range 3–15 years)	8.5

**Table 2:** Summary of themes and main subthemes

Themes	Subthemes
Nurses’ self-care challenges	Utilizing personal protective equipment Personal habits Personal hygiene practices
Patient care challenges	Need for specific interventions Need for psychological support Need for family communication during treatment
Health care delivery system challenges	Personal protective equipment shortages Staffing and volunteer shortages Variety in therapeutic approaches Confusion amid shifting healthcare guidelines

Data analysis showed that the nurses’ self-care included all behavioral approaches to protect them against affliction with COVID-19. Participants believed that the key to patient-care was the self-care, as an effective care provision for patients requires the nurses’ own primary health. With this regard, the three subthemes extracted from the nurses’ speech were: utilizing personal protective equipment, personal habits and personal hygiene practices.

### Utilizing personal protective equipment

According to participants, the use of protectives was the first obsession after the spread of the disease. Protectives include equipment and tools that guarantee nurses’ health in the face of coronavirus. A participant noted: *“…when working in the Covid-19 ward, it is essential to wear specialized protective clothing to safeguard oneself from contracting the virus …” (Nurse 5).* In this regard, one of the participants said: *“…nurses’ mind is more at rest when protective devices such as N95 masks are available when taking care of patients …” (Nurse 2)*. However, participants mentioned some of the existing issues with using protective equipment. One participant described this as *“… wearing heavy and bulky turnout clothing while working in the Covid-19 ward restricts my body movement and comfort …” (Nurse 3)*. Another participant mentions: *“…When you wear an N95 mask and at the same time you have scrubs and a shield, it is so hard to breathe…” (Nurse 11).*

### Personal habits

One of the subthemes identified from the participants’ description of self-care was personal habits. Personal habits refer to unique behaviors that nurses exhibit unconsciously during the care-taking process in the hospital environment. The majority of participants reported being challenged to change or quit these habits when caring for patients with COVID-19. As mentioned by one of the participants:*” … normally, I would have a snack during my shift. However, given the current situation, it is necessary to wash my hands and remove my mask multiple times, which is challenging when eating. Therefore, I made the decision to stop having a snack during my shift … “(Nurse 1).* Another participant shared the experience as: *“…I used to call the babysitter every two hours, but now I avoid doing so due to the risk of infection from their mobile phone or hands…” (Nurse 13)*.

### Personal hygiene practices

The data analysis revealed that the most effective way to prevent the transmission of the disease from an individual to the surrounding environment, and vice versa, was by washing hands after removing gloves and prior to any procedure, or sanitizing them with soap or alcohol. One participant said:*” … after completing most tasks, I should take off gloves and wash my hands…” (Nurse 8)*. Based on the participants’ experiences, it can be inferred that they washed their hands at a higher frequency. As mentioned by one of the participants*” …every time I take off my mask, I should wash my hands…” (Nurse 1)*.

### Patient care challenges

Although most of the participants considered self-care in the process of caring for COVID-19 patients, the significance of addressing both the overt and covert needs of patients in providing care was noteworthy and recurrently mentioned in the interviews. According to the participants’ experiences, the role of nurses in addressing the needs of patients with complex medical conditions is crucial, encompassing need for specific interventions, need for psychological support and need for family communication during treatment.

### Need for specific interventions

The data analysis revealed that participants were providing special attention and support to patients with severe illnesses. The participants emphasized that patients with underlying illnesses, who require more serious attention, need special care from the nursing staff. One participant mentioned: *“… patients with chronic obstructive pulmonary disease (COPD) have inadequate lung function, … severely low oxygen saturation and decreased consciousness…they should be monitored regularly and may require supplemental oxygen therapy.…” (Nurse 17)*, and another participant noted: *“… as a nurse in the COVID-19 ward, I frequently observed coughing as a common clinical symptom … I regularly check the blood pressure of patients with underlying high blood pressure and a persistent cough… (Nurse 11).*

### Need for psychological support

The data analysis revealed that providing care for COVID-19 patients requires attention to psychological support. The participants noted that underlying disease and old age can lead to anxiety. One of the participants said, *“… a 42-year-old female patient with sickle cell disease experienced high levels of anxiety, leading to shortness of breath. She required substantial psychological support…” (Nurse 2).* Another participant mentions: *“… I have seen anxiety and restlessness more in people above 50 years of age … they even directly asked us whether they would die or not…” (Nurse 12).* The experiences of the participants showed that seeing the death of a patient can cause anxiety in other patients. As mentioned by one of the participants: *“… patients may feel anxious when they see activities such as pulling paravans or bringing an emergency trolley to a patient’s bedside … as this event can manifest the death process, even if it does not lead to the patient’s death…” (Nurse 7).*

### Need for family communication during treatment

The data analysis revealed that one of the issues faced by patients during hospitalization was the inability to be with their family and relatives. One participant stated: *“…patients feel worse when they are separated from their families … video calls or phone calls can make a huge difference in how patients feel during their stay…for example, a 17-year-old girl felt much better after making a video call on her mother’s cell phone…” (Nurse 6)*. Another participant mentions:*” … most of our patients didn’t comply with medication or treatment because they missed having their family by their side during hospitalization…” (Nurse 3).*

### Health care delivery system challenges

The study findings underscore challenges within the healthcare delivery system when it comes to providing care for COVID-19 patients. Within this theme, four subthemes emerged: personal protective equipment shortages, staffing and volunteer shortages, variety in therapeutic approaches, and confusion amid shifting healthcare guidelines. These subthemes collectively contribute to the complexity and difficulties faced by nurses in delivering effective care.

### Personal protective equipment shortages

According to the data, the lack of equipment was more concerned with personal protection for the nurses themselves during the care provision. One of the participants in this regard said:*” … we did all we could for patients… they received the required care and services, but we ourselves did not have enough scrubs, shields and specially masks…” (Nurse 5)*. Similarly, another participant stated: *“… at the peak of the disease outbreak in this province, there was a significant shortage of N95 masks… the shortage was so severe that nurses had to resort to using expired masks… despite the risk, nursing staff continued to wear these masks to protect themselves as much as possible while caring for patients …” (Nurse 4).*

### Staffing and volunteer shortages

All participating nurses acknowledged the shortage of required staff in the Covid-19 wards during the outbreak of COVID-19. For example, a participating nurse stated, *“… when the Covid-19 wards were expanded, there was a lack of workforce… we were forced to work there, which was accompanied by many problems…” (Nurse 13)*. Another participant in this regard mentioned:*” …the unknown nature of the virus caused fear among the nursing staff when it first emerged… everyone was too stressed and fearful to volunteer … there was a shortage of volunteers willing to work in the COVID-19 ward…” (Nurse 9)*.

### Variety in therapeutic approaches

In the light of the key points extracted from the interviews, the COVID-19 pandemic has presented a significant challenge for nursing staff. A crucial aspect of managing this disease is identifying the appropriate type of therapy to use at different stages of the disease. The unknown nature of the disease caused confusion among nurses, leading to contradictions in clinical work and further conflicts. In this regard, one of the participants mentioned: *“… there were different therapeutic approaches … some physicians only believed in treating the symptoms… the therapeutic process was conducted based on symptom management alone … others initially prescribed any medication that had shown effectiveness in other parts of the world…” (Nurse 7).*

### Confusion amid shifting healthcare guidelines

Analysis of the participants’ experiences revealed concerns about the transmission of COVID-19, which is highly contagious. One participant mentioned: *“… every day, new instructions were issued … one recommendation advocating for simple masks and another later mandating the use of N95 masks… this caused confusion among nursing staff who were already struggling to care for patients in challenging conditions … the conflicting directives were disheartening … “(Nurse 8)*, and another participant found the confusion in giving instructions to be offensive and believed that it was responsible for disputes in the workplace, said:*”… when we were told that simple masks were sufficient and were working with those masks on, we observed physicians coming with N95 masks to quickly visit patients from the station before rushing out… this created confusion as to which approach was correct… were we supposed to change masks, or was our safety considered less important than that of the physicians? … “(Nurse 4).*

## Discussion

The research presented here offers valuable insights into the experiences and challenges faced by nurses caring for COVID-19 patients. The study, revealed three main themes, each with its own set of subthemes. The key themes revolved around nurses’ self-care, patient care, and the challenges within the healthcare delivery system. These findings shed light on the multifaceted nature of nursing during a pandemic, emphasizing the need for comprehensive support and solutions.

The experiences of nurses providing care during the Covid-19 pandemic have revealed that a major concern is the inadequate protection and improper use of equipment. In accordance with Saffari et al. (2020) [14], the main concern of nurses in dealing with the highly contagious Covid-19 is the availability of adequate protective equipment to ensure their own health and safety.

Although the significance of using personal protective equipment is well-known, Corley’s results from Australia showed that its use can be distressing to healthcare personnel [36].

It is concerning that healthcare personnel face challenges in wearing protective clothing, such as scrubs and masks, and the need for frequent washing and changing of clothes. Additionally, prolonged use of masks can lead to scarring on the face, as noted by Fathi et al.(2020) [37]. Furthermore, this equipment hinders face-to-face contact with both patients and colleagues, which can result in increased fatigue, exhaustion, and hunger among nurses [38, 39].

The use of personal protective equipment can also be exhausting for healthcare personnel, as it can take a considerable amount of time to put on and wear such clothing [40]. To address this issue, breaks can be added during work shifts, working hours can be reduced, personnel can be empowered, and fatigue can be diminished [41].

In addition to the challenge of limited equipment, inadequate guidelines regarding the proper use of personal protective equipment present another obstacle for nursing staff [20]. As healthcare providers are essential resources for a country, their health is of utmost importance not only for consistent and safe care provision but also for controlling the spread of disease. Therefore, all necessary measures should be taken to fully support them, including regular and intensive education to promote preparedness and efficiency among all healthcare providers [8].

Attention must be paid not only to the physical health but also to the psychological well-being of nurses. Healthcare providers who are responsible for caring for Covid-19 patients often experience frustration and emotional distress due to factors such as high work pressure, a large number of patients, fear, stress, and anxiety towards patients and their family members [39].

Lai et al.(2020) [4] found that healthcare personnel experienced significant mental pressure due to overwork, inadequate equipment, and the lack of definitive medication. Given this challenging and unstable situation, it is crucial to prioritize the physical and psychological well-being of healthcare staff by providing them with adequate protection, education, and support [42].

An important point relevant to patient care, as highlighted by the present findings, is the importance of frequent hand-washing to prevent the spread of disease. Previous research has shown that the rate of hand-washing among nurses after providing care is only 25–35% [43]. However, the present study suggests that nurses are now more aware of the seriousness of the condition and the need for regular hand-washing to prevent infection.

The present findings reveal that the disease has both physical and psychological effects on patients, including concerns about their families. Nurses also experience loneliness and dissatisfaction due to the constraints imposed by the disease on their relationships with others. These findings are consistent with Jia et al.(2021) [44], who found that patients not only suffer from physical symptoms but also often feel lonely due to isolation and a lack of contact with family members. Patients who have lost a family member due to Covid-19 may also be pessimistic about therapeutic attempts and care providers. Additionally, Rahmatinejad et al.(2020) [45] reported that patients experience stress, anxiety, low quality of life, fear of death, depression, loneliness, and excitement induced by quarantine or family-related events.

The lack of personal protective equipment and medical staff, particularly nurses, is a major problem in the face of the disease. Wong et al. also identified the shortage of human resources, including nurses, as a major concern during the peak of the H1N1 flu pandemic [46]. Consistent with the present findings, a body of related literature has shown that nurses’ work hours and workload have increased by 1.5-2 times compared to before the pandemic [39, 47, 48].Additionally, the inadequate size of nursing staff and the large number of patients have disrupted adequate care provision and lowered the quality of care services. Therefore, it is necessary to increase the size of the healthcare staff in proportion to the number of patients to reduce the workload and fatigue of personnel [45].

The nurses’ experience of caring for Covid-19 patients revealed a theme of confusion regarding the various types of therapies and methods of virus transmission. This is consistent with a grounded theory study by Kim (2021) [49], which found that the adaptation process of nurses caring for Covid-19 patients comprised periods of confusion. According to Fathi et al.(2020).

 [37], nurses and medical staff may experience confusion due to a lack of adequate knowledge and expertise in the nature of the disease, insufficient experience, confusion about the type of therapy, and challenges in predicting the rate of contagion and number of affected patients. Moreover, the absence of a specific medicine or vaccine for disease prevention and control is a major concern for nurses caring for Covid-19 patients. This uncertainty can have a negative impact on the precision and skill of nurses in providing effective healthcare services [50–52].

Kim et al. (2018) reported that.

The results of Kim et al.‘s (2018) study on nurses’ experiences caring for patients with SARS, H1N1, MERS, and Covid-19 revealed that nurses often lack precise knowledge of care provision instructions and protective facilities, leading to stress and fear [40]. Nurses who cared for MERS and Covid-19 patients in Saudi Arabia reported a fear of inadequate knowledge of therapeutic methods and ways of transmitting the disease [53]. The experience of nurses and medical staff working in ICU during the H1N1 pandemic revealed the difficulty of wearing protective clothing for long hours, ways of controlling contagion, fear of transmitting the disease, and the staff’s spirits in the face of challenges and experience of serving patients [48].

Additionally, the medical staff has been confused and pessimistic about the use of personal protective equipment and the accuracy of infection control protocols due to frequent changes in infection control protocols [40]. However, it is important to note that nurses’ experiences may vary in different parts of the world due to differences in resources, healthcare systems, and cultures. Nevertheless, it is noteworthy that the World Health Organization provides the latest research findings to all countries and those in charge to increase medical staff’s awareness.

## Conclusions

In light of the experiences, concerns, and challenges faced by nurses and medical staff caring for Covid-19 patients, it is essential to prioritize the needs and concerns of healthcare providers to establish a safe and secure healthcare system that is adequately prepared and responsive to the emergence of similar diseases. Moreover, it is necessary to implement strategies to protect healthcare providers from both physical and mental pressures. The experiences of healthcare providers can be used to combat similar infectious diseases and national disasters more effectively in the future.

### Limitations

While this study offered valuable insights, there are notable limitations to this study.

First, the study exclusively focused on the perspectives of nurses, yet including perspectives from other healthcare professionals would offer a more comprehensive understanding. Second, although the study achieved data saturation with its sample size, it remained limited in scope. Expanding the sample to encompass a broader cross-section of nurses could provide a more diverse range of perspectives. Third, due to the nature of outbreak prevention and control, the study couldn’t conduct focus group interviews and collect data from multiple centers to mitigate the risk of cross-infection. Lastly, it is worth noting that this study was conducted over a short-term period. Examining the long-term experiences of the subjects could yield valuable findings.

### Electronic supplementary material

Below is the link to the electronic supplementary material.

Supplementary Material 1

## Data Availability

The datasets used and/or analyzed during the present study are available from the corresponding author upon reasonable request.

## References

[1] Chen S, Yang J, Yang W, Wang C, Bärnighausen T (2020). COVID-19 control in China during mass population movements at New Year. The Lancet.

[2] Yang A, Qiu Q, Kong X, Sun Y, Chen T, Zuo Y, Yuan D, Dai W, Zhou J, Peng A (2020). Clinical and epidemiological characteristics of COVID-19 patients in Chongqing China. Front Public Health.

[3] Tavakoli A, Vahdat K, Keshavarz M (2020). Novel coronavirus Disease 2019 (COVID-19): an emerging Infectious Disease in the 21st Century. Iran South Med J.

[4] Lai C-C, Shih T-P, Ko W-C, Tang H-J, Hsueh P-R (2020). Severe acute respiratory syndrome coronavirus 2 (SARS-CoV-2) and coronavirus disease-2019 (COVID-19): the epidemic and the challenges. Int J Antimicrob Agents.

[5] Gorbalenya AE, Baker SC, Baric RS, de Groot RJ, Drosten C, Gulyaeva AA, Haagmans BL, Lauber C, Leontovich AM, Neuman BW. Severe acute respiratory syndrome-related coronavirus: The species and its viruses–a statement of the Coronavirus Study Group. *BioRxiv* 2020.

[6] COVID-19. Epidemiological Update – 29 September 2023.

[7] Almomani MH, Khater WA, Akhu-Zaheya LM, Alloubani A, AlAshram SA, Azab M, Al-Malkawi AK (2022). Nurses’ experiences of Caring for patients with COVID-19: a qualitative study. Sage Open.

[8] Liu Q, Luo D, Haase JE, Guo Q, Wang XQ, Liu S, Xia L, Liu Z, Yang J, Yang BX (2020). The experiences of health-care providers during the COVID-19 crisis in China: a qualitative study. The Lancet Global Health.

[9] Zhou P, Huang Z, Xiao Y, Huang X, Fan X-G (2020). Protecting Chinese healthcare workers while combating the 2019 novel coronavirus. Infect Control Hosp Epidemiol.

[10] Kellogg MB, Schierberl Scherr AE, Ayotte BJ (2021). All of this was awful: exploring the experience of nurses caring for patients with COVID-19 in the United States. Nurs Forum.

[11] Khajeh Hosseini S, Sayadi A, Mobini Lotfabad M, Heidari S (2020). The effect of Benson’s relaxation technique on sleep quality among shift-working nurses in hospitals. J Hayat.

[12] Abedini S, Takhti HK, Abedini S. Assessing nursing curriculum: graduate nurse viewpoints. Physiology 2011, 13(20.26).

[13] Zakerian SA, Mosaferchi S, Sepidarkish M, Nasiri Z, Yaseri M (2018). The role of individual effective factors on nurses’ job performance. A case study: selected hospitals in Tehran. Occup Med Q J.

[14] Saffari M, vahedian-azimi A, Mahmoudi H (2020). Nurses’ experiences on self-protection when caring for COVID-19 patients. J Military Med.

[15] Sampaio F, Sequeira C, Teixeira L. Nurses’ Mental Health during the Covid-19 outbreak: a cross-sectional study. J Occup Environ Med 2020, 62(10). 10.1097/JOM.0000000000001987 32769803

[16] Sun N, Wei L, Shi S, Jiao D, Song R, Ma L, Wang H, Wang C, Wang Z, You Y (2020). A qualitative study on the psychological experience of caregivers of COVID-19 patients. Am J Infect Control.

[17] González-Gil MT, González-Blázquez C, Parro-Moreno AI, Pedraz-Marcos A, Palmar-Santos A, Otero-García L, Navarta-Sánchez MV, Alcolea-Cosín MT, Argüello-López MT, Canalejas-Pérez C (2021). Nurses’ perceptions and demands regarding COVID-19 care delivery in critical care units and hospital emergency services. Intensive and Critical Care Nursing.

[18] Gualandi R, Ivziku D, Caruso R, Di Giacinto C, Lommi M, Tartaglini D, De Benedictis A. Nurse-Patient Communication and Relationship When Wearing Personal Protective Equipment: Nurses’ Experience in a COVID-19 Ward. *Healthcare (Basel)* 2023, 11(13). 10.3390/healthcare11131960 PMC10340558 37444794

[19] Galehdar N, Toulabi T, Kamran A, Heydari H (2021). Exploring nurses’ perception of taking care of patients with coronavirus Disease (COVID-19): a qualitative study. Nurs Open.

[20] Catania G, Zanini M, Hayter M, Timmins F, Dasso N, Ottonello G, Aleo G, Sasso L, Bagnasco A (2021). Lessons from Italian front-line nurses’ experiences during the COVID-19 pandemic: a qualitative descriptive study. J Nurs Manag.

[21] Boned-Galan A, Lopez-Ibort N, Gil-Lacruz AI, Gascón-Catalán A (2023). Stress impact of COVID-19 in nurse managers. Heliyon.

[22] Wu YC, Chen CS, Chan YJ (2020). The outbreak of COVID-19: an overview. J Chin Med Assoc.

[23] Lai J, Ma S, Wang Y, Cai Z, Hu J, Wei N, Wu J, Du H, Chen T, Li R (2020). Factors Associated with Mental Health Outcomes among Health Care workers exposed to Coronavirus Disease 2019. JAMA Netw Open.

[24] Galehdar N, Toulabi T, Kamran A, Heydari H (2020). Exploring nurses’ perception about the care needs of patients with COVID-19: a qualitative study. BMC Nurs.

[25] Pourgholam N, Zareiyan A, Farsi Z, Abbasiyan K (2023). Exploring perceived stress from caring for coronavirus Disease (COVID-19) patients in nurses: a qualitative study. J Res Nurs.

[26] Shahoei R, Nemati SM, Valiee S (2022). Exploring the experience of nurses in providing care to patients with COVID-19: a qualitative study. J Nurs Res.

[27] WHO Coronavirus (COVID-19.) Dashboard https://covid19.who.int/region/emro/country/ir.

[28] Khanjarian F, Sadat-Hoseini AS (2021). Lived experiences of nurses providing altruistic care to patients with COVID-19. Nurs Outlook.

[29] Chegini Z, Arab-Zozani M, Rajabi MR, Kakemam E (2021). Experiences of critical care nurses fighting against COVID-19: a qualitative phenomenological study. Nurs Forum.

[30] Rourke S, Dimech A, Bacon R, Paterson C (2024). The lived experiences of critical care nurses during the COVID-19 pandemic. A qualitative systematic review. Intensive and Critical Care Nursing.

[31] Elo S, Kääriäinen M, Kanste O, Pölkki T, Utriainen K, Kyngäs H (2014). Qualitative content analysis: a focus on trustworthiness. SAGE Open.

[32] Corcoran L, Friedenreich CM, McNeely ML, Culos-Reed NS, Bell G, Dickau L, Courneya KS, Vallance JK (2023). A qualitative study examining newly diagnosed Breast cancer patients’ experiences of participating in the Alberta moving beyond Breast Cancer (AMBER) prospective cohort study. BMC Cancer.

[33] Tong A, Sainsbury P, Craig J (2007). Consolidated criteria for reporting qualitative research (COREQ): a 32-item checklist for interviews and focus groups. Int J Qual Health Care.

[34] Braun V, Clarke V (2021). To saturate or not to saturate? Questioning data saturation as a useful concept for thematic analysis and sample-size rationales. Qualitative Res Sport Exerc Health.

[35] Graneheim UH, Lundman B (2004). Qualitative content analysis in nursing research: concepts, procedures and measures to achieve trustworthiness. Nurse Educ Today.

[36] Corley A, Hammond NE, Fraser JF (2010). The experiences of health care workers employed in an Australian intensive care unit during the H1N1 Influenza pandemic of 2009: a phenomenological study. Int J Nurs Stud.

[37] Fathi E, Malekshahi Beiranvand F, Hatami Varzaneh A, Nobahari A (2020). Health Care workers challenges during Coronavirus Outbreak: the qualitative study. J Res Behav Sci.

[38] Maben J, Bridges J (2020). Covid-19: supporting nurses’ psychological and mental health. J Clin Nurs.

[39] Sun N, Wei L, Shi S, Jiao D, Song R, Ma L, Wang H, Wang C, Wang Z, You Y (2020). A qualitative study on the psychological experience of caregivers of COVID-19 patients. Am J Infect Control.

[40] Kim Y (2018). Nurses’ experiences of care for patients with Middle East respiratory syndrome-coronavirus in South Korea. Am J Infect Control.

[41] Aein F, Delaram M (2014). Giving bad news: a qualitative research exploration. Iran Red Crescent Med J.

[42] Huang C, Wang Y, Li X, Ren L, Zhao J, Hu Y, Zhang L, Fan G, Xu J, Gu X (2020). Clinical features of patients infected with 2019 novel coronavirus in Wuhan, China. Lancet.

[43] Garus-Pakowska A, Sobala W, Szatko F (2013). Observance of hand washing procedures performed by the medical personnel after the patient contact. Part II. Int J Occup Med Environ Health.

[44] Jia Y, Chen O, Xiao Z, Xiao J, Bian J, Jia H (2021). Nurses’ ethical challenges caring for people with COVID-19: a qualitative study. Nurs Ethics.

[45] Rahmatinejad P, Yazdi M, Khosravi Z, Shahisadrabadi F (2020). Lived experience of patients with coronavirus (Covid-19): a phenomenological study. J Title.

[46] Wong ELY, Wong SYS, Kung K, Cheung AW, Gao TT, Griffiths SM (2010). Will the community nurse continue to function during H1N1 Influenza pandemic: a cross-sectional study of Hong Kong community nurses?. BMC Health Serv Res.

[47] Rana W, Mukhtar S, Mukhtar S (2020). Mental health of medical workers in Pakistan during the pandemic COVID-19 outbreak. Asian J Psychiatr.

[48] Corley A, Hammond NE, Fraser JFJI. The experiences of health care workers employed in an Australian intensive care unit during the H1N1 Influenza pandemic of 2009: a phenomenological study. 2010, 47(5):577–85. 10.1016/j.ijnurstu.2009.11.015 PMC7125717 20036360

[49] Kim J, Kim S. Nurses’ Adaptations in Caring for COVID-19 Patients: A Grounded Theory Study. *Int J Environ Res Public Health* 2021, 18(19). 10.3390/ijerph181910141 PMC8507730 34639445

[50] Nogee D, Tomassoni AJ (2020). Covid-19 and the N95 respirator shortage: closing the gap. Infect Control Hosp Epidemiol.

[51] Shrestha GS (2020). COVID-19 pandemic: shortage of Personal Protective Equipment, Use of Improvised surrogates, and the Safety of Health Care Workers. J Nepal Health Res Counc.

[52] Peterson CJ, Lee B, Nugent K. COVID-19 vaccination hesitancy among Healthcare Workers-A Review. Vaccines (Basel) 2022, 10(6). 10.3390/vaccines10060948 PMC9227837 35746556

[53] Khalid I, Khalid TJ, Qabajah MR, Barnard AG, Qushmaq IA (2016). Healthcare workers emotions, perceived stressors and coping strategies during a MERS-CoV outbreak. Clin Med Res.

