# Supplementary material for: Through the eyes of nurses: a content analysis of nurses’ experiences in caring for COVID-19 patients
Source: BMC Nurs. 2023 Nov 15;22:431. doi: 10.1186/s12912-023-01601-5 (PMC10647166; doi:10.1186/s12912-023-01601-5)
Supplement: Supplementary file 1 — Supplementary Material 1 [file 12912_2023_1601_MOESM1_ESM.docx]

**Interview Questions**

The main question:

Could you please share your experience in caring for patients with COVID-19?

Questions asked based on the participants’ responses and the interview guide:

- How did you feel when caring for COVID-19 patients?
- What were the biggest challenges you faced while caring for COVID-19 patients?
- What were some of the most significant resource deficiencies you encountered while caring for COVID-19 patients, and how did you work around these challenges?
- What kind of precautions did you take to protect yourself and others from COVID-19 while caring for patients?
- What types of confusion and uncertainty arose when caring for COVID-19 patients?
- How did you adapt your approach to patient care to address the unique needs of COVID-19 patients?
